# Lung adenocarcinoma relapse with emerging EGFR mutation following complete response of small cell lung cancer warrants routine re-biopsy: A case report

**DOI:** 10.3389/fonc.2022.1024655

**Published:** 2022-10-26

**Authors:** Minna Zhang, Yi Tang, Junlei Wang, Qian Liu, Bing Xia

**Affiliations:** ^1^ Department of Thoracic Oncology, Affiliated Hangzhou Cancer Hospital, Zhejiang University School of Medicine, Hangzhou, China; ^2^ Department of Medicine, Berry Oncology Corporation, Beijing, China; ^3^ Cancer Center, Zhejiang University, Hangzhou, China; ^4^ Key Laboratory of Clinical Cancer Pharmacology and Toxicology Research of Zhejiang Province, Hangzhou, China

**Keywords:** non-small cell lung cancer, small cell lung cancer, lung adenocarcinoma, histological transformation, EGFR

## Abstract

Transformation of small cell lung cancer (SCLC) to lung adenocarcinoma (LUAD) is rarely reported. Here, we report a case initially presented with SCLC and was diagnosed as LUAD when the lesion relapsed at the same site. A 56-year-old patient with SCLC who received etoposide and cisplatin chemotherapy combined with radiotherapy achieved a complete radiological response. After 28 months of stable disease, a computed tomography scan revealed a new lesion at the same site as the primary tumor. Pathological examination suggested a LUAD with an emerging *EGFR* exon 19 deletion. The patient was then treated with icotinib and achieved a near-complete radiological response. Nineteen months later, the patient developed resistance caused by *EGFR* T790M mutation and received treatment with osimertinib. At the last follow-up in January 2022, the patient was symptom-free. This case warrants re-biopsy and genetic testing as a routine operation when SCLC relapses at the same site as the primary tumor for an extended period, and prospective investigation is required.

## Introduction

Non-small cell lung cancer (NSCLC) transformation to small cell lung cancer (SCLC) following treatment with tyrosine kinase inhibitors (TKIs) or immunotherapy has frequently been reported to be a mechanism of acquired resistance (1, 2). The post-treatment occurrence of histological transformation from NSCLC to SCLC is up to 14% (3), but cases of SCLC transformation to lung adenocarcinoma (LUAD) are rare (4, 5), and the clinical significance and best treatment option underlying such cases are still unknown.

Herein, we report a case of SCLC-LUAD histological change. To our knowledge, this is the first report of *EGFR* mutant LUAD transformed from *EGFR* wild-type SCLC after chemoradiotherapy. We present the following case following the CARE reporting checklist.

## Case report

**Figure 1:**
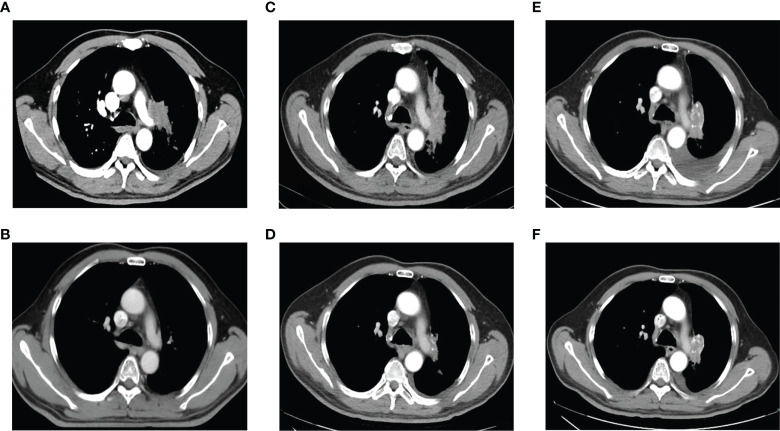
Chest computed tomography at initial diagnosis, during and after treatment. **(A)**. At initial diagnosis. **(B)**. After treatment of EP combined with radiotherapy. **(C)**. At the first relapse. **(D)**. After treatment with chemotherapy plus icotinib. **(E)**. After icotinib resistance. **(F)**. After treatment of osimertinib.

**Figure 2:**
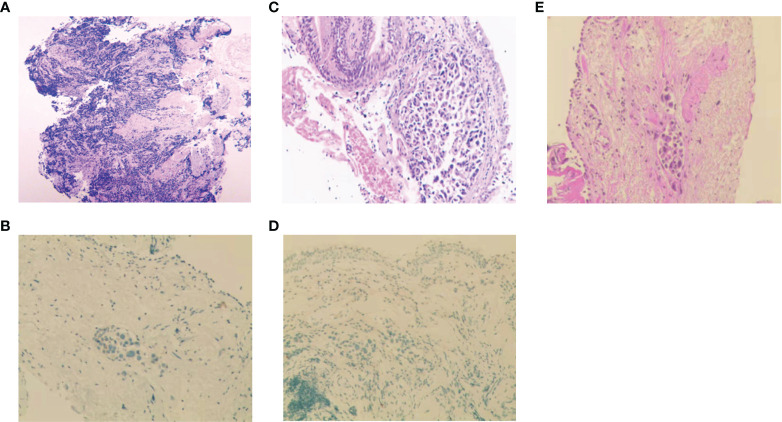
Histopathologic analysis at initial diagnosis, during and after treatment. **(A-D)** H&E staining and IHC analysis of Napsin-A at initial diagnosis **(A, B)**, at the first relapse **(C, D)**. **(E)** H&E staining after icotinib resistance.

**Figure 3:**
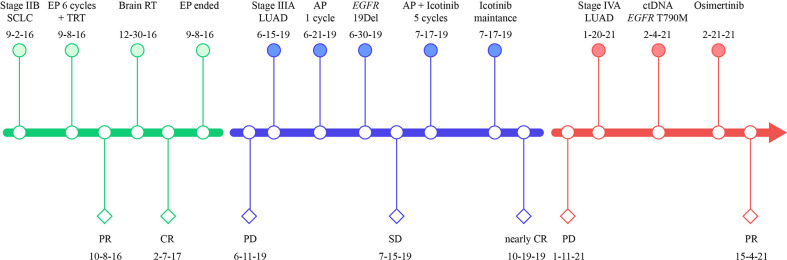
Treatment timeline of our case. SCLC, small-cell lung cancer; LUAD, lung adenocarcinoma; EP, etoposide plus cisplatin; TRT, thoracic radiation therapy; RT, radiation therapy; AP, cisplatin plus pemetrexed; PR, partial response; CR, complete response, PD, progressive disease; EGFR, epidermal growth factor receptor; 19Del, exon 19 deletion.

A 56-year-old man with 30-pack-year smoking history experienced a dry cough and shortness of breath during exercise for one month. He had well-controlled diabetes for eight years by metformin and no significant family history of cancer. Chest computed tomography (CT) revealed a left hilar mass with obstructive pneumonia of the left upper lobe (**Figure 1A**). Hematoxylin-eosin (H&E) staining of the bronchoscopic specimens showed small round and poorly differentiated cells without non-small cells (**Figure 2A**). Immunohistochemistry (IHC) staining showed that the biopsy was negative for Napsin-A, a marker for LUAD (**Figure 2B**). Taken together, this patient was diagnosed with left central SCLC (T3N0M0, limit-stage, stage IIB, according to the eighth edition American Joint Commission on Cancer classification criteria) (**Figure 3**). A targeted comprehensive genomic profiling (CGP), which contains 654 cancer-related genes, was performed on the tumor tissue by next-generation sequencing (NGS). The CGP assay detected 18 mutations, such as *TP53* (c.159G>A, p.W53*, 75.8%) and *RB1* (c.2239dupG, p.E747Gfs*4, 70.4%) (**Table 1**). Concomitant inactivation of p53 and RB, which is nearly universally in SCLC, is deemed as an essential initiating molecular event (6, 7). The patient, an employee with national medical insurance, was not religious and actively cooperated during the diagnosis.

**Table 1:** Genetic variants in the case revealed by the CGP assasy.

	Gene	Variant type	Exon	HGVSc	HGVSp	VAF/CNV
SCLC	*BARD1*	SNV	4	c.1178G>T	p.G393V	51.95%
	*BCR*	SNV	16	c.2954A>C	p.K985T	31.43%
	*CCND3*	SNV	5	c.732G>T	p.Q244H	76.15%
	*FANCA*	SNV	23	c.2149A>T	p.M717L	64.29%
	*MERTK*	SNV	15	c.2021T>C	p.M674T	19.29%
	*NF1*	SNV	33	c.4420G>A	p.A1474T	45.92%
	*POT1*	SNV	7	c.220A>G	p.K74E	18.74%
	*RB1*	Insertion	22	c.2239dupG	p.E747Gfs*4	70.44%
	*RPTOR*	Deletion	30	c.3574_3582 delGTCTACGAC	p.V1192_D1194 delVYD	12.88%
	*TP53*	SNV	4	c.159G>A	p.W53*	75.84%
	*CALR*	CNV	NA	NA	NA	4.62
	*CDK4*	CNV	NA	NA	NA	4.95
	*GNA13*	CNV	NA	NA	NA	4.78
	*IRS2*	CNV	NA	NA	NA	72.98
	*KLF5*	CNV	NA	NA	NA	63.79
	*MCL1*	CNV	NA	NA	NA	4.62
	*PPM1D*	CNV	NA	NA	NA	4.77
	*RPTOR*	CNV	NA	NA	NA	4.59
First Relapse	*EGFR*	Deletion	19	c.2240_2257 del	p.L747_P753 delinsS	2.22%
	*TP53*	SNV	10	c.1010G>C	p.R337P	4.26%
Second Relapse	*EGFR*	Deletion	19	c.2240_2257 del	p.L747_P753 delinsS	0.57%
	*EGFR*	SNV	20	c.2369C>T	p.T790M	0.62%

CGP, comprehensive genomic profiling; HGVS, Human Genome Variation Society; VAF, variant allel frequency; CNV, copy number variation; NA, not available.

Six cycles of etoposide and cisplatin (EP) concurrent with thoracic radiotherapy followed by prophylactic cranial irradiation were given to the patient (etoposide: 120 mg/m^2^ for the first 2 cycles, 100 mg/m^2^ for the rest of 4 cycles, on days 1-3; cisplatin: 75 mg/m^2^ days 1-3; chest radiation therapy: 55Gy/25F, concurrently with the 3-4 cycles of EP treatment; brain radiation therapy: 25Gy/10F, between the fourth and fifth cycles of EP treatment), and resulted in a complete radiological response (CR), according to the Response Evaluation Criteria in Solid Tumors version 1.1 (**Figure 1B**, **3**). The patient showed good tolerance to chemoradiotherapy with a transient gastrointestinal response.

Twenty-eight months after CR, the patient was readmitted with a dry cough. A CT scan revealed a new lesion at the same site of the primary tumor (**Figure 1C**). H&E staining of the bronchoscopic re-biopsy displayed adenoid structure (**Figure 2C**). Napsin-A was positive in the IHC analysis (**Figure 2D**). The results of CT, H&E staining, and IHC suggested the diagnosis with left central LUAD (T4N0M0, stage IIIA). The CGP assay on bronchoscopic biopsy revealed two mutations, *EGFR* (c.2240_2257del, p.L747_P753delinsS, 2.22%) and *TP53* (c.1010G>C, p.R337P, 4.26%) (**Table 1**). Based on the diagnosis, he was given the first-generation EGFR-TKI icotinib plus chemotherapy (pemetrexed: 500 mg/m^2^, days 1; cisplatin: 75 mg/m^2^, days 1-3; icotinib: 125 mg, t.i.d., p.o.) (**Figure 3**). After six cycles of chemotherapy plus icotinib followed by icotinib maintenance, the patient achieved a near-complete radiological response (**Figure 1D**, **3**).

After 19 months of stable disease, a chest CT disclosed a mass occupying the left upper hilar portion with obstructive pneumonia (**Figure 1E**). Histopathological analysis of the bronchoscopic re-biopsy reported LUAD structure (**Figure 2E**). Taken together, the diagnosis of left central LUAD was confirmed (T4NXM1, stage IVA) (**Figure 3**). *EGFR* T790M was detected in blood plasma by the NGS CGP assay. The patient began to take osimertinib (80mg, q.d., p.o.) and achieved a partial response (**Figures 1F**, **3**).

During the 6-year treatment period, the patient adopted the best self-care and went to the outpatient clinic monthly for further consultation. Any changes in medication and symptoms were recorded, and there were no uncontrolled adverse events.

All procedures performed in this study involving human participants were in accordance with the ethical standards of the institutional review board of Hangzhou Cancer Hospital and with the Helsinki Declaration (as revised in 2013). Written informed consent was obtained from the patient for publication of this case report and accompanying images. A copy of the written consent is available for review by the editorial office of this journal.

## Discussion

Transformation to LUAD from SCLC is rarely reported (4, 5, 8–10) Abeloff et al. documented the emergency of non-small cell components by H&E staining in autopsies of SCLC patients (4). Wang et al. reported a case of SCLC-LUAD transformation after the initial tumor vanished (5). Morinaga et al. described a case of LUAD with *EGFR* 19Del. The patient underwent LUAD-SCLC-LUAD pathological change with the same EGFR mutation (10). Sequist et al. presented a LUAD patient with *EGFR* L858R, who also underwent a pathological change of LUAD-SCLC-LUAD with *EGFR* L858R maintained (9). Takagi et al. recorded a case who underwent sequential LUAD, SCLC and LUAD pathological change with *EGFR* L861Q retained (8) To the best of our knowledge, our case firstly reported SCLC-LUAD transformation with emerging *EGFR* 19Del.

Ouadah et al. suggested that neuroendocrine cells are stem cells that can give rise to alveolar type 2 cells (11). Oser et al. suggested that activation of EGFR signaling could be essential for the fully differentiated alveolar-cell phenotype, and SCLC could resume adenocarcinoma histology when the EGFR signaling was restored (3). And in our case, in addition to the IHC biomarker, *EGFR* E19Del was detected when the transformation to LUAD occurred after CR following EP treatment, which may explain the SCLC-LUAD change.

It should be noted that the diagnosis was based on biopsy, H&E staining, and IHC. However, a biopsy is subjected to spatial selection bias due to intratumor heterogeneity. In this case, there was no shared mutations between the initial and relapsed tumor tissues in the targeted CGP assay by NGS (**Table 1**). Although the treatment with EP combined with radiotherapy was very effective, and the patient achieved a complete radiological response, we could not rule out the probability of a mixed histologic type in the initial tumor, in which case there might be a mixture of SCLC and LUAD in the initial diagnosis, but SCLC was predominant. After chemoradiotherapy, adenocarcinoma gradually became prominent, with SCLC dwindling.

It is also possible that the LUAD might be a metachronous primary tumor since the genetic variations detected in the SCLC and LUAD biopsy tissues of this patient were utterly different (**Table 1**). However, LUAD is more commonly localized peripherally than in a central location. The emerging LUAD appeared at the same site of the original lesion (both by imaging and bronchoscopy) significantly lowers the possibilities of metachronous primary tumor.

Patients with extensive-stage SCLC usually respond well to first-line chemoradiotherapy, but the resistance often develops within 6 months (12). However, few patients have extended progression-free survival over two years (13). Chen et al. reported that patients with *TP53* mutation or more than five mutations have more prolonged progression-free survival to first-line chemotherapy (14). In this case, the patient with SCLC had 18 mutated genes, including *TP53* and *RB1* which are nearly universally inactivated in SCLC (6).

Although icotinib, approved by the National Medical Products Administration, is a standard of care in China for EGFR mutant NSCLC (15, 16), icotinib plus chemotherapy is not yet. However, the co-mutation of TP53 (17, 18) and low frequency of EGFR mutation (4.26%) (19)of the patient might dampen the benefit of EGFR TKI alone. Meanwhile, the result of phase 3 study NEJ009 suggested EGFR TKI plus chemotherapy might benefit patients with EGFR patient (20). In addition, our case had shown good response and tolerance to chemoradiotherapy. Taken together, the patient was given the first-generation EGFR-TKI icotinib plus chemotherapy.

## Conclusion

In conclusion, we reported a rare case with histological evolution from *EGFR* wild-type SCLC to *EGFR* mutant LUAD. Re-biopsy and genetic testing provided a more accurate diagnosis, which guided the choice of subsequent precise treatments, therefore, it should be recommended as a routine operation when SCLC relapses at the same site of the primary tumor after a long remission period.

## Data availability statement

The datasets presented in this article are not readily available because of ethical/privacy restrictions. Requests to access the datasets should be directed to the corresponding author.

## Ethics statement

Written informed consent was obtained from the individual for the publication of any potentially identifiable images or data included in this article.​

## Author contributions

BX contributed to the conception and design and provided administrative support. MZ contributed to treat the patient, analysis and wrote the manuscript. YT and JW analyzed the data. All authors contributed to the article and approved the submitted version.

## Conflict of interest

Author JW is, and QL was employed by Berry Oncology Corporation Beijing, China.

The remaining authors declare that the research was conducted in the absence of any commercial or financial relationships that could be construed as a potential conflict of interest.

## Publisher’s note

All claims expressed in this article are solely those of the authors and do not necessarily represent those of their affiliated organizations, or those of the publisher, the editors and the reviewers. Any product that may be evaluated in this article, or claim that may be made by its manufacturer, is not guaranteed or endorsed by the publisher.
